# “Sequencing Matters”: Investigating Suitable Action Sequences in Robot-Assisted Autism Therapy

**DOI:** 10.3389/frobt.2022.784249

**Published:** 2022-03-09

**Authors:** Kim Baraka, Marta Couto, Francisco S. Melo, Ana Paiva, Manuela Veloso

**Affiliations:** ^1^ School of Computer Science, Carnegie Mellon University, Pittsburgh, PA, United States; ^2^ Group on AI for People and Society (GAIPS), INESC-ID, Porto Salvo, Portugal; ^3^ Instituto Superior Técnico, Universidade de Lisboa, Porto Salvo, Portugal; ^4^ Centro de Desenvolvimento da Criança, Hospital Garcia de Orta, Almada, Portugal

**Keywords:** socially assistive robots, autism therapy, action selection, storytelling, attention skills, personalization, human-robot interaction

## Abstract

Social robots have been shown to be promising tools for delivering therapeutic tasks for children with Autism Spectrum Disorder (ASD). However, their efficacy is currently limited by a lack of flexibility of the robot’s social behavior to successfully meet therapeutic and interaction goals. Robot-assisted interventions are often based on structured tasks where the robot sequentially guides the child towards the task goal. Motivated by a need for personalization to accommodate a diverse set of children profiles, this paper investigates the effect of different robot action sequences in structured socially interactive tasks targeting attention skills in children with different ASD profiles. Based on an autism diagnostic tool, we devised a robotic prompting scheme on a NAO humanoid robot, aimed at eliciting goal behaviors from the child, and integrated it in a novel interactive storytelling scenario involving screens. We programmed the robot to operate in three different modes: diagnostic-inspired (*Assess*), personalized therapy-inspired (*Therapy*), and random (*Explore*). Our exploratory study with 11 young children with ASD highlights the usefulness and limitations of each mode according to different possible interaction goals, and paves the way towards more complex methods for balancing short-term and long-term goals in personalized robot-assisted therapy.

## 1 Introduction

Autism Spectrum Disorder (ASD) is a set of developmental conditions that affect an individual’s social abilities, verbal and non-verbal communication, and potentially motor and cognitive skills (APA 2013). In past years, the introduction of robots in therapy for children with ASD has gained increased interest (Diehl et al., 2012; Scassellati et al., 2012; Thill et al., 2012; Cabibihan et al., 2013; Huijnen et al., 2017). Socially assistive (humanoid) robots offer a number of characteristics that make them attractive tools for use in ASD therapy. They are *predictable*, which can help reduce the anxiety that some children may experience when navigating the uncertainty of a social interaction; they are *engaging*, which can allow for richer and more sustained interactions during therapy; and they are *simplified social models* of humans, which allows children to explore a more basic version of social interactions before applying their learned skills to interactions with people. This paper considers a robot-assisted autism therapy scenario specifically targeting attention skills, a major area of impairment for young children with ASD (APA 2013). These attention skills include the ability to direct one’s attention from one object to another when prompted, and constitute a crucial developmental milestone needed to unlock more complex social abilities throughout development (e.g., Baron-Cohen (1989); Mundy et al. (1990); Murza et al. (2016)).

Due to the high variability across different children with ASD and the high level of uncertainty when interacting with them, autism therapists heavily rely on personalization strategies. Every therapy session is different depending on the ASD profile of the child, their responsiveness to given stimuli, and their level of engagement with the task at hand (Schaaf and Roley 2006). While therapists engage in such strategies naturally, thanks to years of professional experience, the challenges in applying these principles to effective robot-assisted therapeutic tasks are numerous. These challenges include:

• *Assessment*—Building useful profiles of children interacting with robots consists in assessing features characterizing their interaction with the robot. This is a challenging goal for several reasons.

First, children’s response to robots may significantly differ from their response to humans (Bekele et al., 2014). As a result, there might not be a systematic way to predict response to a robot given data on interaction with a human. Second, the cost of exploration may be high. Individuals with ASD are often extremely sensitive to details, and a single “wrong step” in the robot’s behavior may result in serious consequences, such as jeopardizing the willingness of the child to interact again with the robot. Finally, the amount of data that a robot can collect with a specific child is limited, which makes it difficult to estimate, from scarce data, child features that are useful for the interaction.

In this work, we base our feature assessment method on standard diagnostic procedures widely used by human therapists.

• *Personalization methods*—Personalization of actions selected during a social interaction plays a crucial role in the context of autism therapy, but is still lacking in socially assistive robotics research. The most effective approach in human-administered therapy is to tailor the “just-right challenge” to each individual (Schaaf and Roley 2006). Effectively, this strategy translates into finding the right balance between making social cues “easy enough” to limit task duration and maintain engagement, but “hard enough” to promote improvement over time by challenging current child abilities.

In this work, our personalization strategy in mode Therapy aligns with typical strategies followed by human therapists that have been shown to promote learning in the long-term.

• *Integration in naturalistic context*—Since most ASD therapy tasks rely on aspects of social interaction, it is necessary to integrate them in an engaging scenario with a consistent context and progression. Maintaining stable engagement levels with such a population is particularly challenging and also particularly helpful as it reduces uncertainty in the robot’s ability to predict children’s responses.

In this work, we integrate structured tasks of interest within a larger interactive storytelling scenario.

While different methods for personalization have been investigated across a variety of therapeutic tasks and scenarios, three main aspects are overlooked in the existing socially assistive robotics literature (see Section 2). First, most personalization methods don’t explicitly account for the fact that children will often require several trials by the robot before they respond appropriately (according to the pre-defined task goal). Also, the methods that do support multiple robot trials don’t investigate how different sequencing methods for consecutive robot actions may affect interaction-related or therapeutic metrics. Second, existing personalized action selection methods don’t take into account the value of *variability* (loosely, how diverse actions in a given sequence are). We hypothesize that variability, while not necessarily serving a therapeutic purpose, may play an important role in maintaining the fluency of the interaction. Third, it remains unclear whether and how diagnostic information (obtained through child-therapist interaction) can inform child-robot interaction. This paper attempts to fill these three gaps by first reasoning about action *sequences* instead of individual actions for each instance of a task; second, by controlling for different levels of variability within these sequences across the different modes (Assess, Therapy, Explore); and third, by including diagnostic information as part of the empirical analysis of the study.

In this paper, we report on an exploratory study whereby a NAO humanoid robot engages with 11 children with different ASD severities in a storytelling scenario. Our scenario integrates structured tasks inspired by the Autism Diagnostic Observation Schedule (ADOS) (Lord et al., 2012), the gold standard tool for ASD diagnosis. The study, run in collaboration with a child development center at a Portuguese hospital, aimed at analyzing the role of action sequencing on children’s response to the robot’s actions. While the actions available to the robot within the structured tasks were pre-defined, we controlled the *action sequences* that the robot executed within different instances of the same task. In particular, we considered three modes of operation for the robot, corresponding to three different ways of generating action sequences. The first mode, *Assess*, is inspired by the ADOS procedure within the tasks of interest. The second mode, *Therapy*, uses the profile assessed in the previous mode to generate action sequences inspired by the way therapists would select their actions in alignment with therapeutic goals. The third mode, *Explore*, generates completely random action sequences.

The rest of the paper is organized as follows. After discussing related work (Section 2), we detail our use of the ADOS tool for developing a set of prompting actions and modes in structured tasks on a NAO humanoid robot (Section 3). Section 4 explains the integration of these tasks in an interactive storytelling scenario involving the robot and two controllable screens. Section 5 reports on our empirical study of children’s behavioral responses during a session with the robot. We proceed to discussing our findings in Section 7 and end with conclusions and future work in Section 8.

## 2 Related Work

This section presents an overview of related work in robot personalization and attention-related tasks, identifying the research gap in existing research. Furthermore, as adjacent but relevant topics to situate our approach, we briefly mention relevant work on robot assessment of social child behavior, and interactive storytelling, with a focus on the ASD domain.

### 2.1 Robot Personalization and Adaptation in the Autism Spectrum Disorder Domain

Personalization can be understood as a special case of general adaptation (changing an agent’s behavior as a function of different conditions). Personalization means that the robot adapts its behavior according to inter-individual differences. In contrast, in what follows, we will use the term “adaptation” to specifically refer to intra-individual adaptation, whereby a robot changes its behavior to account for changes in an individual’s behavior over time.

While personalization in human-robot interaction has been an increasingly relevant topics across several different domains (Ahmad et al., 2017; Rossi et al., 2017), in the autism domain many existing approaches still heavily rely on tele-operation or user-driven content customization (Palestra et al., 2016). According to Esteban et al. (2017), higher levels of autonomy are needed to bootstrap the performance and flexibility of such systems. The authors believe that supervised autonomy would be the ideal solution, leveraging the advantages that autonomy has to offer while including the therapist in the loop to ensure that the robot does not perform detrimental actions. The authors also developed a platform-independent architecture for automatic personalization (Cao et al., 2018) of robot behavior. On the perception side, the personalization of algorithms for detecting child behaviors, for instance related to affect and engagement, has also been investigated (Rudovic et al., 2018). These works highlight the importance of personalization in every component of a system developed for ASD intervention.

Other major aspects of socially assistive autonomy are real-time and long-term adaptation. Examples of adaptive systems in the autism context include an affective robot adaptation method through multimodal measurements of affect to regulate a basketball-based task (Conn et al., 2008), and a model for graded robotic cueing in an imitation task (Greczek et al., 2014). Recent work by Clabaugh et al. (2019) demonstrated the effectiveness of their personalization and adaptation approach for in-home robot-assisted therapy. In our work, the robot adjusts its action level when no success is observed, which can be seen as an example of basic adaptation.

Other methods for long-term adaptation, including changing exercise difficulty based on assessed skill or past performance have been investigated in therapy and tutoring settings (Leyzberg et al. (2014); Scassellati et al. (2018)), however it is still unclear how personalization can be integrated as soon as the first session, or how the concept of “difficulty” can be also transferred to social cues rather than problem-solving tasks. It is also still unclear to date how personalized robot behavior should balance therapeutic goals and other objectives of the interaction, such as maintaining engagement.

### 2.2 Attention-Related Robotic Tasks

Attention skills are a major area of impairment of ASD children as such an impairment deprives them of social information from an early age, resulting in a disruption of their development (Mundy and Neal 2000). A significant attention impairment relates to joint attention, which pertains to the ability to coordinate attention between a social partner and an object or event, allowing two people to share awareness of those objects or events (see e.g., Dawson et al. (2004)). One example of joint attention behavior is to follow someone’s eye gaze or follow someone’s pointing towards a target object. Following the direction of someone’s gaze emerges early in the development and it is seen a facilitator of social learning, particularly language (Mundy and Newell 2007). Following someone’s gaze and paying attention to what they are looking at, helps children organize and process unstructured social situations (Mundy and Newell 2007). Mundy et al. (2007) found that, after controlling for general cognition, the frequency with which children engage in joint attention relates to important developmental aspects such language acquisition, IQ, social competence and self-regulation.

Given the importance of joint attention in children’s development, it is not surprising that this is a targeted behavior for therapy in ASD children who show significant impairments in this area (Baron-Cohen 1989). As such, several works have looked at robot-mediated solutions to train joint attention skills of children with ASD. In particular, Anzalone et al. (2014) investigated a spatio-temporal model of response to robots versus humans, showing generally lower response to robots. Furthermore, the Michelangelo project (Anzalone et al., 2019) tested three protocols for eliciting joint attention in Typically Developing (TD) children and children with ASD, in a setup similar to the one in our work. As expected, the authors found that TD children responded to joint attention prompts more frequently. The same authors also found improvement on children with ASD responses to joint attention following a 6 month training protocol, which shows promise and the usefulness of using a robot to train joint attention in children with ASD. Other work in this space has analyzed gaze patterns in different gaze orienting tasks (Edmunds et al., 2017) as well as non-verbal cognitive tasks (Kunda et al., 2016).

Robotic interventions for joint attention do show promise, however, the extent to which they can be integrated in a successful therapy plan needs additional research. For example, Cao et al. (2019) tested responsive joint attention for TD children and children with ASD in two conditions: elicited by a human and elicited by a robot. Overall the results show that despite an increased interest in the robot’s face, children spent more time looking at targets in the human condition. This does not mean that robots do not have an important role and should not be used to train joint attention but it does raise a question of how robots can improve joint attention skills of children with ASD. The choice of behaviours and protocols seems to have a crucial role in the design of a robotic interaction aimed at improving joint attention skills.

The work that comes closest to this work is that of Warren et al. (2015), who developed ADOS-inspired robotic tasks involving screens as targets for joint attention prompts. In our work, we use a similar setup, but with different research questions. While their focus was on studying the effect of fixed action sequences throughout multiple sessions, our focus is to study and compare alternative action sequences within a session. Our study also goes a step further by integrating the setup in a naturalistic storytelling interaction, considering additional tasks/prompts, providing a validation of response coding (see Section 6.1), and looking at a larger sample size.

### 2.3 Robot-Administered Assessment

In addition to therapy, perhaps the most investigated use of robots in relation to autism is assessment for the sake of diagnosis. The idea is to take advantage of the objectivity and controllability of robots to avoid the variability and subjectivity of human-administered assessment.

The work in robot-administered diagnosis has considered several subproblems, such as developing quantitative metrics of social response (Scassellati 2005), developing standardized tasks inspired by existing diagnostic tools (Petric et al., 2014), or researching algorithms for relevant robot perception (Kokot et al., 2018; Presecan et al., 2018). Petric et al. (2017) developed a framework for robot-administered diagnosis based on a Partially Observable Markov Decision Process (POMDP) formulation, to assess specific child features, using robot perception of multiple social and communication cues. They developed four robotic tasks inspired by the ADOS, and tested them in a clinical setting (Petric et al., 2014).

In this work, one of the robot’s modes of operation uses a similar approach to robot control to estimate a profile of the child for the purpose of subsequent personalization, and not for precise diagnosis. In our previous work (Baraka et al., 2019), we provided an alternative role for robots in the diagnosis process by using the robot as a simulation platform for complementing the diagnostic training of therapists. We believe that the human aspect of diagnosis is an important one, as ultimately an evaluation of ASD should be measured with respect to a human rather than an artificial agent.

### 2.4 Robot Storytelling

While not central to our research goals, the use of storytelling is an important part of our work that supports the integration of the tasks of interest into a more naturalistic context. We therefore provide a brief overview of related work on robot storytelling.

Storytelling has often been used in human-robot interaction and technology-based scenarios, with both adults and children, for a wide array of educational goals (Tartaro 2006; Dillon and Underwood 2012; Fridin 2014; Kory Westlund et al., 2017). However, the interactive component of these scenarios remains limited, and has been the focus of recent investigation (Paradeda et al., 2017; Park et al., 2017; Ligthart et al., 2020). In particular, Sun et al. (2017) introduced a collaborative storytelling scenario in which both the robot and the child contribute to create a story. While most storytelling scenarios focus on the expressivity of the robot and the educational goals, in this work we introduced novel ways of introducing interactive engagement in robotic storytelling, through the use of screens that illustrate and support the story with engaging video snippets.

### 2.5 Summary and Research Contributions

Due to the highly interdisciplinary nature of socially assistive robotics as a research area, the relevant literature appears to be scattered across contributions in robot behavior or interaction design, interactive algorithms, and empirical contributions. This paper’s main contribution to the field is *empirical*, in the sense that it investigates the effectiveness of different action sequences on child response across a spectrum of ASD severities, in order to inform the development of automatic personalization mechanisms in the future. The paper’s secondary contribution is *integrative*, meaning that our approach builds on and integrates a unique set of elements to serve the main contribution. These elements are: 1) the use of tasks that target a major impairment in individuals with ASD, namely joint attention, prevalent in previous socially assistive robotics work, 2) an approach to action sequence design grounded in psychological tools and therapist expertise, and 3) a focus on naturalistic interaction in the study design, through interactive storytelling as a way to maintain children engaged across multiple task instances.

In the following sections, we delve into our research methodology.

## 3 Autism Diagnostic Observation Schedule-Inspired Robotic Prompting Scheme

This section describes our robotic prompting scheme developed for a NAO humanoid robot, and inspired by the structure of the ADOS tool (Lord et al., 2012). The ADOS is composed of 10 standardized tasks as well as a number of features used to code different aspects of behavior observed during the administration session. The two robotic tasks considered in this research are inspired by the “algorithmic” nature (clear instruction steps and if/then conditions) of two ADOS tasks, related to joint attention and response to name calling. After describing the interaction setup considered, we present our developed robotic actions inspired by these ADOS tasks. We then discuss our flexible robot control architecture, which allows for different modes of operation (namely Assess, Therapy, and Explore).

### 3.1 Interaction Setup

**FIGURE 1:**
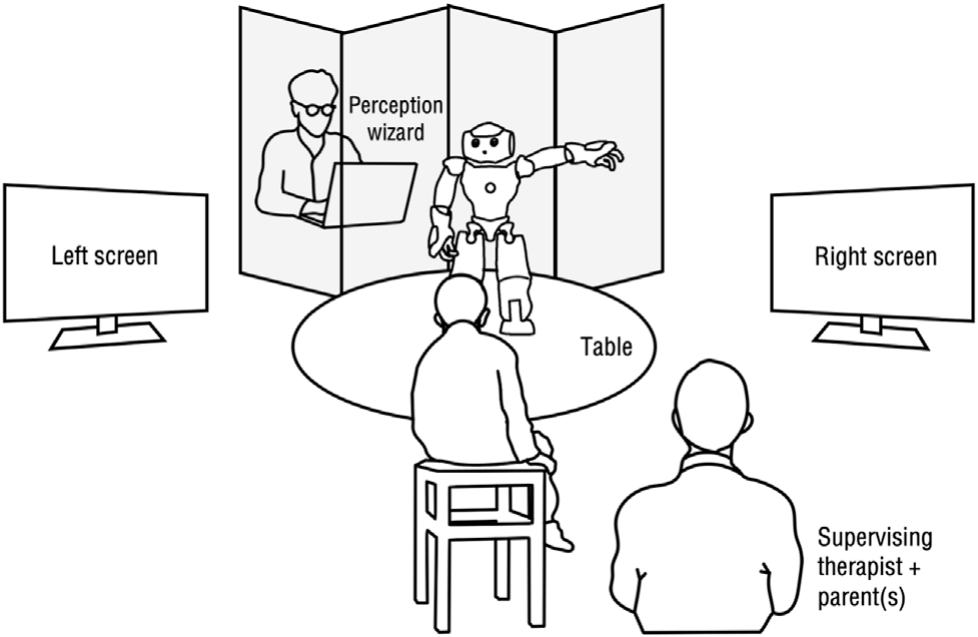
Interaction setup. Figure is only meant for illustrative purposes; relative positions and sizes of the components are not exact.

Figure 1 shows the physical setup used in this paper, inspired by the work of Warren et al. (2015) who demonstrated its suitability for young children with ASD. We found this scenario to be attractive to explore the idea of personalization of attention-related interactions, as it allows for both control and flexibility when compared to scenarios involving physical objects, portable digital devices (e.g., tablets) (Bernardo et al., 2016), or scenarios where the child moves around the space (Melo et al., 2018). The setup consists of a NAO robot standing on a table, at which the child is seated, and two 49.4 cm LCD screens positioned at around a 90° angle on both sides of the child’s chair.

The robot engages in two main tasks of focus, inspired by the ADOS:

• “Joint Attention” task (JATT) — The robot directs the child’s gaze from looking at the robot to looking at a target screen where a video will play.
• “Name Calling” task (NAME) — The robot directs the child’s gaze from looking at the video on one of the screen back to looking at the robot.

A “perception Wizard” provides the robot with information about the child’s gaze behavior through a computer interface, hidden behind a single-sided mirror at an angle that maximizes the view to the scene. Specifically, during each of the two tasks, the Wizard is responsible for triggering a “success” event whenever the child performs the goal behavior for that task (i.e., orienting their gaze in the right direction). For the JATT task, a success triggers a short video snippet. For the NAME task, a success stops the video playing on the screen where the child is looking. While eye-tracking or head-tracking technology were available for us to use, we preferred to rely on human perception, as such technologies are too invasive and inaccurate, especially for children with attention impairments who tend to move considerably. Furthermore, it allows us to focus on the action selection problem, while factoring out the additional noise that comes with an automated perception system.

A single processing unit allows the control of each screen individually. The Wizard’s machine runs the main software to automatically control the behavior of both the robot and the screens, while allowing the Wizard to provide success information when needed. A wired network connection through a switch between all computing units was used to minimize delays and connectivity issues. We used the Thalamus framework (Ribeiro et al., 2012) to facilitate communication between the distributed modules. For safety purposes, the robot’s feet were stuck to the table using tape to avoid falls, as we have noticed that some children were particularly keen on touching and poking the robot.

Next we describe the actions that we programmed the robot to execute during the two tasks.

### 3.2 Action Scales

As part of the ADOS tasks, there exist systematic “algorithms” for evaluating a child’s response to joint attention and response to name, through scales of actions with increasing levels of explicitness (“hierarchies of presses” in ADOS terminology). Each action corresponds to a more or less explicit action taken by the therapist with the common aim of eliciting a goal behavior on the child’s part. The ADOS actions and the goal child behaviors are summarized in columns 2–4 of Table 1.

**TABLE 1:** Summary of our robotic actions, organized along a scale with increasing levels of explicitness (1–4), inspired by the actions of ADOS tasks.

Task	Level	ADOS action	Robot action
	1	Shift + “[Name], look!”	(Gaze + speech) Shift gaze from child to target screen + “[Name], look!” (static picture on both screens)
JATT (Goal behavior: Look at target)	2	Shift gaze + “[Name], look at that!”	(Gaze + speech + point) Shift gaze + “[Name], look at that!” + point (static picture on both screens)
	3	Shift gaze + “[Name], look at that!” + point	(Gaze + speech + point + video) Shift gaze + “[Name], look at that!” + point + play muted video on target screen (static picture on other screen)
	4	Activate target (toy)	(Gaze + speech + point + video + sound) Shift gaze + “[Name], look at that!” + point + play video with localized sound on target screen (static picture on other screen)
	1	“[Name!]”	“[Name!]”
NAME (Goal behavior: Look at provider)	2	Ask parent/caregiver to call name	“[Name], look over here!”
	3	Ask parent/caregiver to make a familiar sound	“[Name], look over here!” + blink lights
	4	Ask parent/caregiver to do whatever necessary to get child’s attention	“[Name], look over here!” + blink lights + wave arm

Inspired by the structure of the ADOS tasks, we developed similar action scales for the robot, aiming to elicit the corresponding goal behavior from the child. Column 5 of the table summarizes our developed robotic actions. We should point out that the aim was not to replicate the content of the ADOS actions with high fidelity. Rather, we came up with similar scales adapted to our scenario and accounting for a range of responses along the scales. Also, to ensure an increasing level of explicitness for the actions, we structured them such that action *a* + 1 is a replica of action *a* with an added element that either adds intensity to the stimulus (e.g., sound on top of video) or facilitates the understanding of the action (e.g., pointing added to gaze). We used the SERA software architecture (Ribeiro et al., 2016) to control the robot’s multi-modal behaviors. Speech was automatically generated by NAO’s built-in text-to-speech engine.

We fine-tuned our actions based on pilot trials with four TD children, two children with ASD, and one child with minimal to no evidence of ASD (according to ADOS). Specifically, for task JATT, we had to take special care with the behavior of the screens, as it seemed from our pilots that the sharp transitions from a black screen to an image or video was a very salient stimulus that transiently overpowered the robot’s role. For this reason, we decided to pre-load a static picture on both screens, corresponding to the first frame of the video to be shown, and to keep the brightness of the screens on a low setting.

### 3.3 Robot Control

**FIGURE 2:**
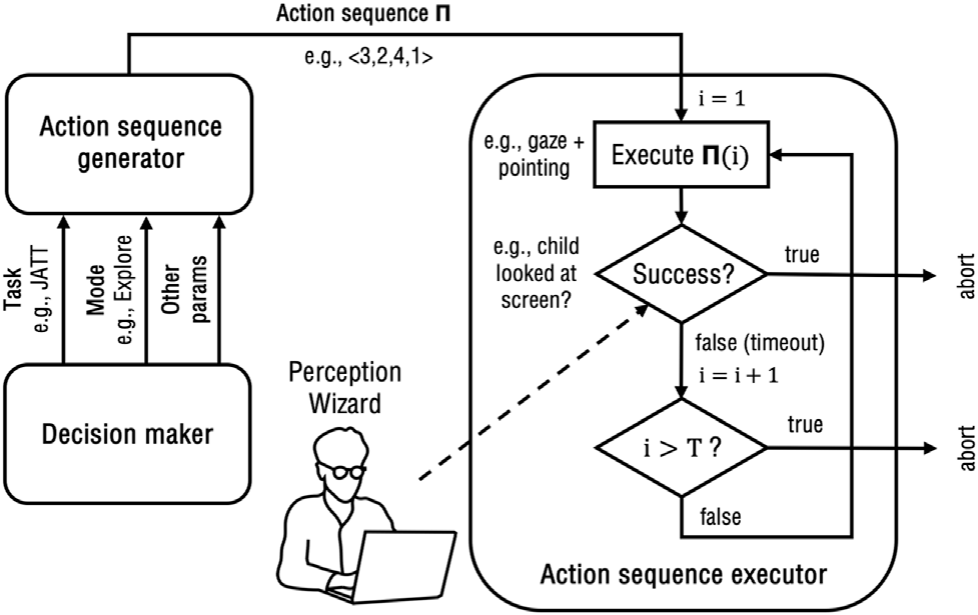
Robot control architecture.

Figure 2 shows the relation between the different modules of the robot control architecture. Before starting the execution of the task, the robot first generates an *action sequence* $\Pi =\left\langle a_{1},a_{2}\ldots ,a_{T}\right\rangle$ , i.e., a plan of actions to be executed over consecutive time steps. An action sequence generation module produces these sequences according to parameters communicated by a high-level decision maker, including task type, robot operation mode, as well as other scenario- and child-related parameters. The action sequence $\Pi =\left\langle 1,3,2,2\right\rangle$ , for instance, means that the robot will perform action of level 1 as a first trial, then potentially execute more trials with actions of level 3, 2, and again 2, until the goal behavior is observed or the sequence is exhausted. While in this work we restrict the action sequence length *T* to 4, our architecture is general enough to allow for arbitrary sequences of any length. An action sequence execution module executes the actions on the robot sequentially, until either a success is triggered by the Wizard or the sequence is exhausted. The trigger of the next action in the sequence is a timeout in case no success occurs. Based on our pilots, we set the duration of the timeout to 3.5 s.

### 3.4 Child Profile Assessment

In the ADOS, the therapist goes through the actions hierarchically from least to most explicit until the expected response is observed, and records the level of the first successful action. This number can be seen as a measure of abnormality of response to the task. In this work, since we consider two tasks, the child profile is represented as a pair of features “Response to Joint Attention (RJA)” and “Response to Name (RNA),” denoted by (RJA,RNA). These features take on the lowest action level (1–4) at which a success is observed in the corresponding task. If none of the four action levels achieve a success, we assign to the corresponding feature a value of 5. In a typical ADOS session, features RJA and RNA are measured only once. In a robotic scenario however, we expect much greater variability in the response due to the novelty effect associated with the robot, as well as the scenario as a whole. For this reason, accurately estimating values of a feature of interest *f* may require several measurements. Given *n* measurements *f* ^(1)^, *f* ^(2)^…, *f* ^(*n*)^, we estimate *f* as:

$$f=\left\{\begin{array}{c} rnd\left(\overset{n}{\underset{1}{\sum }}\frac{f^{\left(i\right)}}{n}\right)\;\text{if}\;\overset{n}{\underset{1}{\sum }}\frac{f^{\left(i\right)}}{n}\mathrm{mod}\;2\neq 0.5\;\\ rnd\left(\overset{n}{\underset{2}{\sum }}\frac{f^{\left(i\right)}}{n-1}\right)\;\;\;\text{if}\;\overset{n}{\underset{1}{\sum }}\frac{f^{\left(i\right)}}{n}\mathrm{mod}\;2=0.5\; \end{array}\right.$$ (1)

where rnd () represents rounding to the nearest integer. In other words, in case of an estimate lying exactly in the middle of two levels, we omit the first sample. The latter is more prone to novelty factors and is hence, in comparison to more recent samples, less reflective of subsequent performance of the child on the task. Eq. 1 applies for estimating both RJA and RNA.Examples (*n* = 4):

$f^{\left(1\right)}=3,f^{\left(2\right)}=3,f^{\left(3\right)}=4,f^{\left(4\right)}=2\to f=rnd\left(\frac{3+3+4+2}{4}\right)=3$

$f^{\left(1\right)}=3,f^{\left(2\right)}=3,f^{\left(3\right)}=2,f^{\left(4\right)}=2\to f=rnd\left(\frac{3+2+2}{3}\right)=2$

### 3.5 Robot Modes

We consider three modes of operation for the robot during task execution. These modes effectively translate into different action sequences, as follows:

• Assess mode—The robot follows the action scale hierarchically, from least to most explicit action, as is done in the original ADOS tasks. The action sequences for this mode are fixed for all children, and of the form $\Pi =\left\langle 1,2,3,4\right\rangle$ . This mode enables the robot to build a profile of the child by recording the lowest action level at which the child responds for the two tasks, as explained in the previous subsection.

• Therapy mode—The robot follows a therapy-inspired action sequence characterized by consistency, repetition, and personalization. For a given child feature *f*, the first two actions in the action sequence are repetitions of action *f*, while the last two actions are repetitions of action *f* + 1. In the edge cases where *f* = 4 or *f* = 5, this mode generates four repetitions of action 4.Examples:

$f=2\to \;\Pi =\left\langle 2,2,3,3\right\rangle$

$f=3\to \;\Pi =\left\langle 3,3,4,4\right\rangle$

$f=4\to \;\Pi =\left\langle 4,4,4,4\right\rangle$ .

This mode was developed in accordance with typical therapeutic strategies, based on the concepts of “just-right challenge” and task grading (Hersch et al., 2005; Schaaf and Roley 2006), as well as a discussion with autism experts.

It is important to mention that the goal of this mode is not to minimize the number of actions needed to observe a success, otherwise the robot could always select the most explicit action 4. Instead, in alignment with therapeutic goals, this mode chooses the least explicit action that has been shown to work on a particular child, while adapting the level to a higher one if no success is observed after the exhaustion of half the sequence. This choice promotes learning (in the long term) at the cost of potentially increasing the number of actions needed for a success to occur.

• Explore mode—The robot follows completely random action sequences, where actions are drawn uniformly and independently at every time step. These action sequences are characterized by inconsistency, unpredictability, and lack of personalization, and therefore have little therapeutic value.

We should point out that in any of the modes presented above, the action sequence represents a plan, whose execution is aborted if a success occurs, i.e., if the child performs the goal behavior. While our robot control architecture allows for more modes than the ones above, those were the ones that best fit our scenario and research goals. Moreover, while in the future one may consider an algorithm that alternates between an Explore phase and an “Exploit” phase, the Therapy mode does not update its action sequence as a function of the outcome of mode Explore.

## 4 Interaction Scenario

In order to test our robotic prompting scheme in the context of an extended social interaction, we designed and implemented an interactive storytelling scenario, in which short excerpts of an animated cartoon on the screens regularly support and illustrate the robot’s speech delivery. The JATT task is repeatedly used throughout the interaction to direct the child’s attention to one of the two screens where the cartoon excerpt is to be shown. Following this task, the robot uses the NAME task to call back the child’s attention and resume the storytelling.

### 4.1 Storytelling Design

The story we chose is based on an episode of a Japanese cartoon, Ox Tales, dubbed in European Portuguese. Popular in previous generations, this amusing cartoon is much lesser known by the current generation of children. This reduces the chances of current children having strong (positive or negative) feelings about it. The episode was selected based on the simplicity of the plot and the presence of simple actions for the child to imitate, which the robot uses to engage the child throughout the story. We transcribed, simplified and rewrote the video episode in a storytelling style with simple language to ensure that children with different language abilities would be able to follow the story. We then edited and adapted the length and organization of the story based on our pilot trials, aiming at optimizing for child engagement, clarity of robot speech, and plot simplicity. In parallel to the verbal content of the story, we extracted and edited 12 cartoon snippets of 12 s each. We handpicked snippets that showed interesting actions throughout the story, including four snippets whose aim is to introduce specific characters of the story.

The robot used its built-in European Portuguese text-to-speech engine for both the storytelling part and the interactive tasks. Even though pre-recorded voice could have been more engaging and natural-feeling for the sake of storytelling, the choice of text-to-speech aligned with our long-term goal of a personalized and adaptive solution that includes modulating speech content automatically. As a result, we opted for the greatest level of reliable autonomy possible on the robot side. To increase the expressivity of the robot during storytelling, we animated it with a “Breathing” behavior consisting of swinging its weight side to side between each leg at a rate of 30 times per min. We also added expressive hand gestures (palm up, randomly alternating between left and right), inspired by simple gestures typically used by storytellers (Ribeiro and Paiva 2012).

### 4.2 Interaction Timeline

**FIGURE 3:**
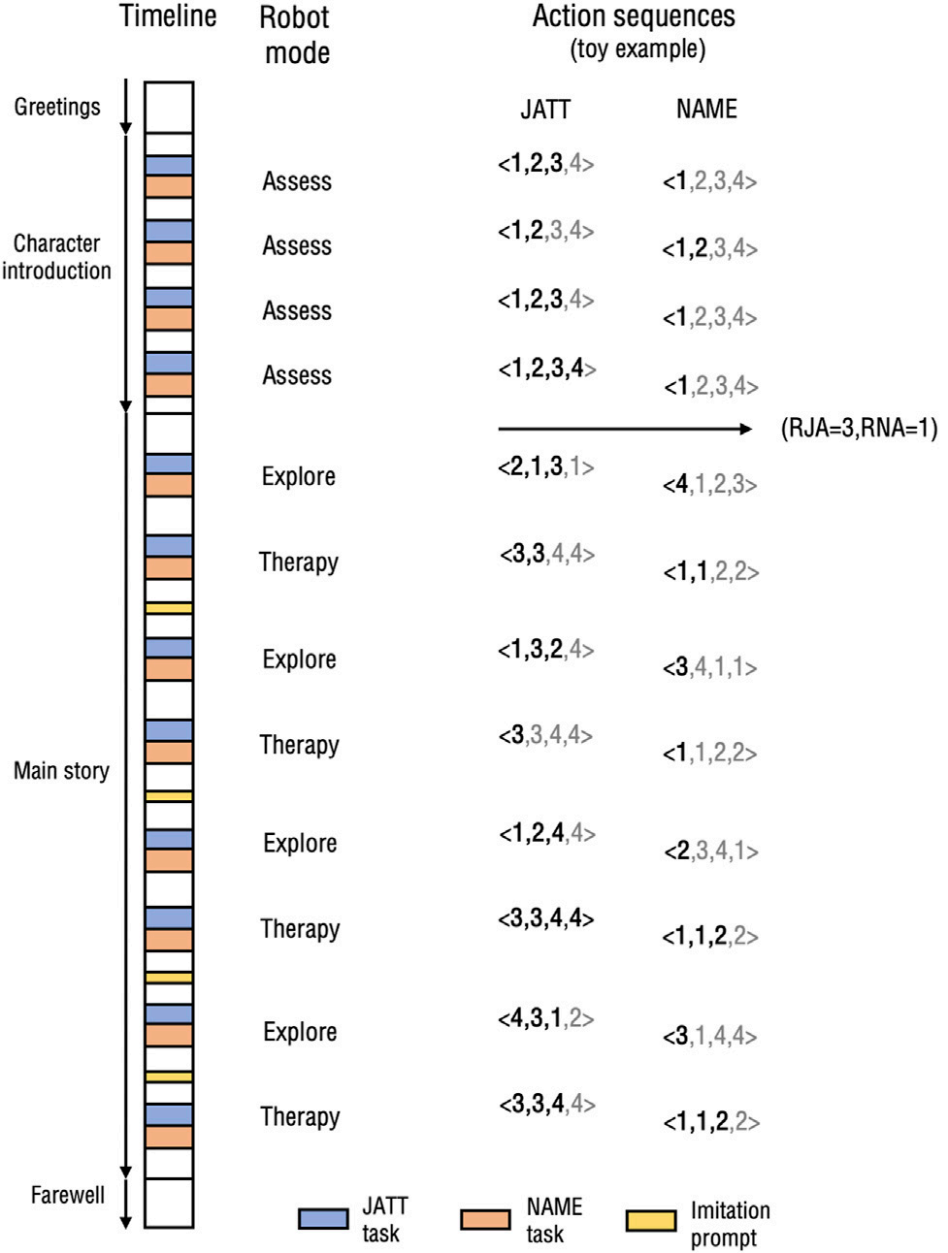
Chronological scenario timeline (to approximate scale) along with corresponding robot modes, illustrated with a toy example. Greyed out portions of action sequences represent planned actions that were not executed due to a success.

Figure 3 shows the timeline of the interaction. The scenario alternated between storytelling and interactive prompting using the two tasks. The robot also used imitation prompts meant to keep the child engaged (imitation ability is also commonly impaired in children with ASD and has been the focus of some robot-assisted interventions (Taheri et al., 2015; Zheng et al., 2015)).

The robot started with some greetings, which consisted in introducing itself and asking for the child’s name until the child responded (or the parent, in case of failure). The robot then moved to the assessment phase, in which it presented four characters of the story, using four instances of each task. After the assessment phase, the robot started the main story phase. In both phases, the robot used the cartoon snippets in the tasks to illustrate relevant story content. We tried to balance the number of words as much as possible between the different story parts defined by the occurrence of the tasks. Any success or timeout in JATT triggered the 12-s video snippet of the corresponding part of the story. Any success or timeout in NAME turned both screens to black for a short period of time, then updated both screens with a new static image corresponding to the next part of the story as the robot resumed its speech. In the imitation prompts, the robot asked the child to imitate a total of four gestures related to the story plot (pretending to fly, pretending to run, covering eyes, and looking around). To further keep the child engaged, throughout the story the robot relied on questions such as “What do you think will happen?,” or “Will Ox Tales be able to fly?” Right before the main story phase, as well as during the farewell phase, the cartoon theme was played with music on both screens.

In the assessment phase, the robot was programmed to be in Assess mode, and performed each task a total of four times. It used the recorded levels at which the children responded to estimate their profile. In the main interaction phase, the robot alternated between the Therapy and Explore modes, performing each task a total of eight times. We remind the reader that the Therapy mode only relied on the result from the assessment phase, and unlike some existing machine learning algorithms that interleave exploration and exploitation in their policies, it was not influenced by the results of the Explore mode.

## 5 Empirical Study

Based on the scenario described in the previous section, we ran an empirical study aimed at analyzing how children with ASD responded to the robot’s different action sequences. The present section provides details about the study participants and experimental procedure.

### 5.1 Participants

We recruited 11 children with different ASD severities from the Child Development Center at the Garcia de Orta Hospital in Almada, Portugal, to participate in the study. These children did not include the seven participants of our pilot trials. The criteria for selection were: between two and 7 years old, and diagnosed with ASD according to the ADOS. In addition to these criteria, we consulted with the therapist working with those children asking whether they thought the child would respond well to this type of scenario (e.g., sitting on a chair for a relatively long period of time). We also asked if there were any factors that may not make them suitable for our scenario (e.g., fear of robots).

The ages of our sample ranged between 2 years 9 months and 7 years 1 month (*M* = 4.64, SD = 1.36 years). Seven were male (63.6%) and four female (36.4%). Three children had low severity scores, six moderate and two severe. Three of the participants (27.3%) had interacted with a robot before (but not NAO) in the context of a separate study. All participants successfully completed the session, except for one who only completed the assessment phase.

### 5.2 Experimental Procedure

One of the experimenters first obtained informed consent from the child’s parent/primary caregiver for using the data collected for research purposes, and optionally to use media for public research communication. Because the date at which the ADOS was administered differed significantly across children, we decided to re-assess the features of interest (RJA and RNA) in an interaction with a human right before the session with the robot. The re-assessment was done by one of the examiners who has experience with children with ASD and has a post-doctoral level training in psychological research. Some children had the ADOS administered the same week the study was run, so we did not reassess them and used the available ADOS features directly.

**FIGURE 4:**
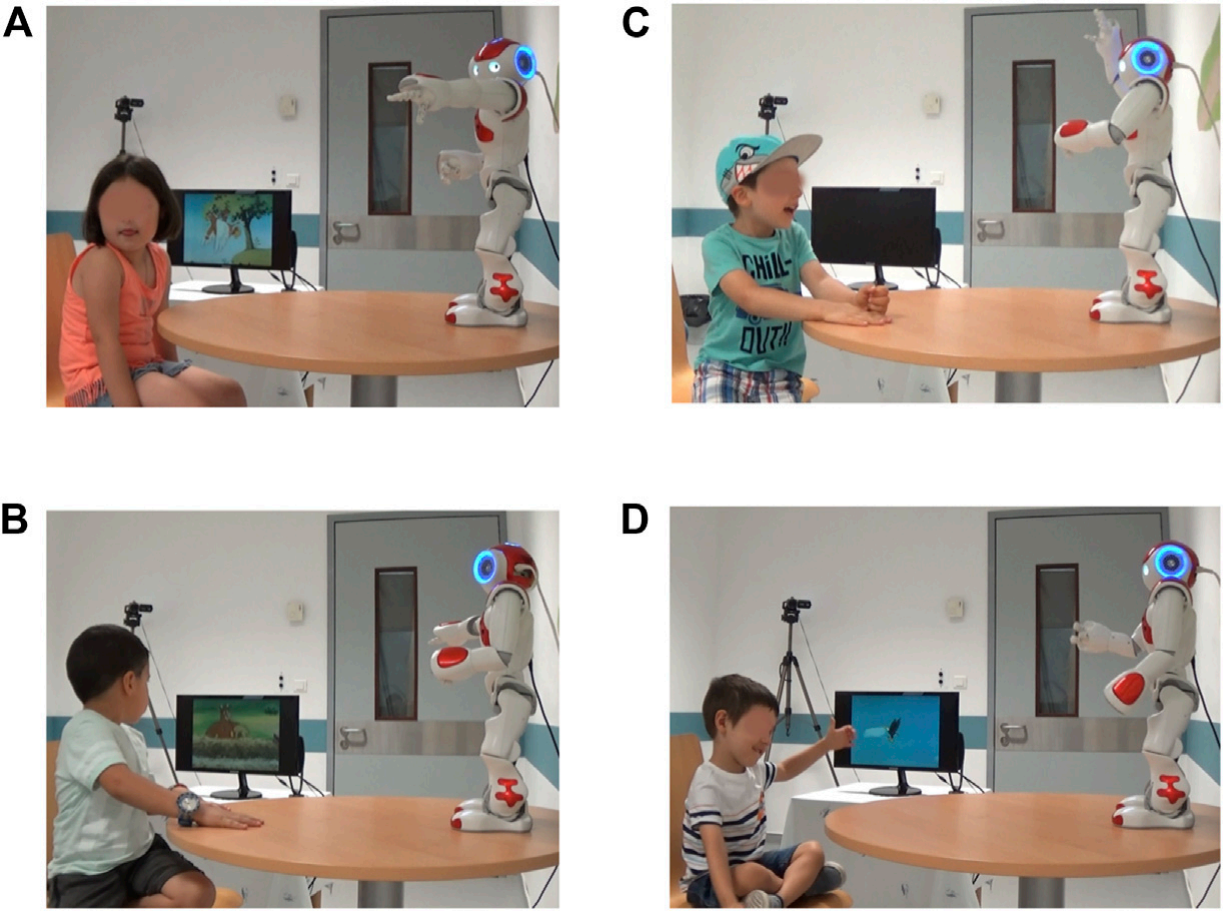
Snapshots from the experimental sessions. **(A)**, **(B)**: JATT successes for the right and left screen respectively (right screen not shown). **(C)**: NAME success for action level 4. **(D)**: Child imitating the robot’s movement as instructed during storytelling for increased engagement. Images are shared under informed consent of parent/primary caregiver.

The experimenter then brought the child into the experiment room, along with their parent(s)/caregiver(s). Before initiating the session, the child was given ample time to explore the robot, and was encouraged to touch it and talk to it. During this initial time, the Wizard controlled the robot progressively using a library of pre-defined actions meant to attract the child in case of lack of interest, or to calm the child in case of fear or distress. After the child was seated and ready to interact with the robot, the semi-autonomous control of the robot was initiated. From there on, the experiment timeline outlined in Figure 3 started. The total session time ranged between 15 and 20 min approximately. Figure 4 shows some snapshots from different sessions.

The parents were instructed to minimally intervene, especially during the tasks, so as not to bias our results. During the tasks, the experimenter followed strict guidelines when intervention was needed. She only intervened to make sure the child was looking at the robot before the robot initiated the JATT task, and at the screen (or at least away from the robot) for the NAME task, both of which are important pre-conditions for the tasks we are studying.

The role of the Wizard was played by a second experimenter during all the sessions. To ensure that there was no bias in the data he provided, we asked an autism therapist, who was agnostic to the aims of the study, to separately record her coding of children’s responses for later comparison. Since she was not familiar enough with the Wizard interface, we decided that it was best for her not to operate the interface directly, as a low latency was crucial when triggering successes.

Throughout the interaction, the robot recorded the responses of each child to each robot action, as either “success” or “failure,” based on the Wizard’s input. This data was then used to compute objective measures to compare the effectiveness of the different modes (see Section 6.3).

### 5.3 Counterbalancing

In the assessment phase, the choice of screen (left/right) in the JATT task alternated between consecutive tasks, and the choice of first screen was counterbalanced across participants. In the main interaction phase, the choice of screen was randomly selected while ensuring equal left/right proportions for each participant and not allowing more than two consecutive instances on the same screen, in order to avoid any practice effect. Also, the choice of the first mode in the alternating sequence (Therapy/Explore) was counterbalanced across participants.

## 6 Results

We extracted all relevant data from the robot logs, and analyzed them using a combination of SPSS, Matlab and Excel software. Our analysis mainly revolved around children’s responses to the action sequences in the different robot modes.

### 6.1 Wizard Coding Method Validation

We computed Cohen’s Kappa interrater agreement between the Wizard’s coding, which dictated the robot’s behavior, and the coding of the autism expert present during the sessions. We compared the ordinal variables representing the index in the action sequence at which a success occurred. If no success occurred after exhaustion of the action sequence, we assigned a value of 5. If a success was triggered by the Wizard but not coded as a valid success by the expert, we assigned to the expert’s coding a unique value (e.g., 0). Our analysis shows a strong agreement between the two raters (*κ* = 0.89, *n* = 248), based on common interpretation guidelines (McHugh, 2012). Based on observation, we believe that disagreements mainly occurred when the children exhibited multiple quick consecutive gaze shifts, which introduced ambiguity in coding.

### 6.2 Assessment Results

**FIGURE 5:**
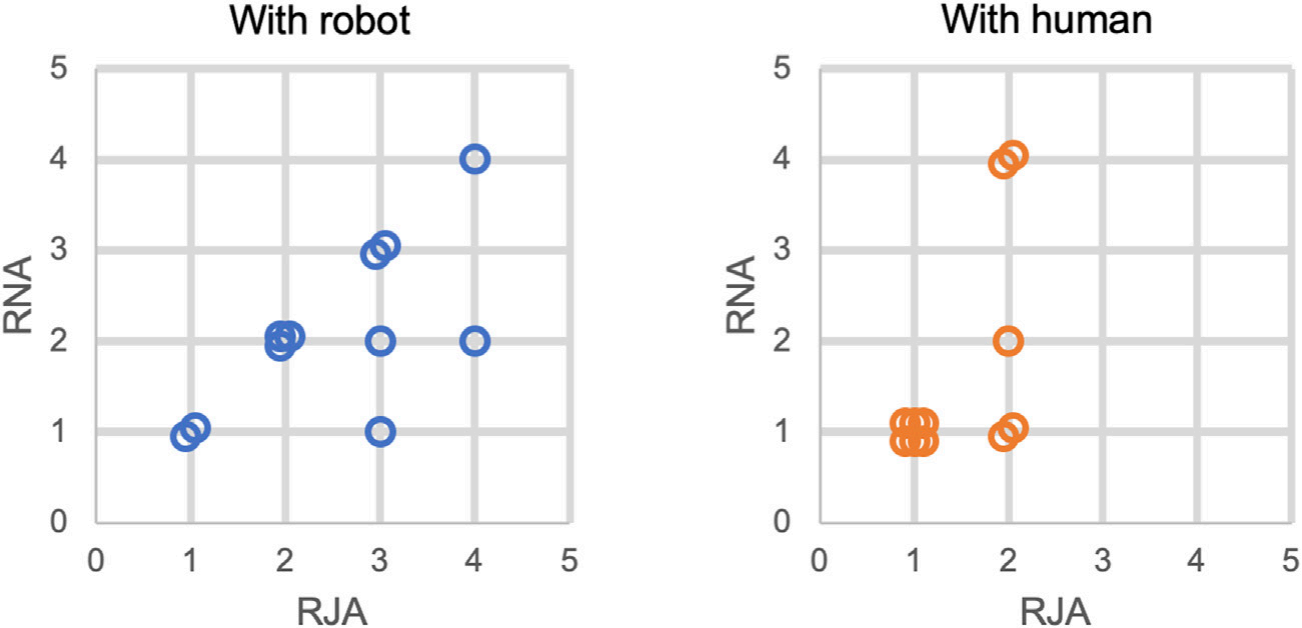
Distribution of children profiles during interaction with the robot and with a human (ADOS assessment) in similar tasks. Overlapping points were slightly disturbed for better visibility. For comparison with actual ADOS feature values, reported values need to be reduced by one unit as ADOS feature values start at 0 by convention.

Figure 5 reports the values of features RJA and RNA as assessed by the robot, according to Eq. 1, and the values of these features according to the human-administrated ADOS assessment (see Section 5.2). Looking at the plots in Figure 5, the immediate observation is a difference in spread. A paired samples Friedman’s two-way analysis of variance by ranks showed that the distributions between robot-assessed and human-assessed features are statistically significantly different (*χ* ^2^ (1) = 11.00**, *p* = 0.001 for RJA, and *χ* ^2^ (1) = 4.46*, *p* = 0.035 for RNA). Similarly, a Spearman correlation test showed no statistically significantly correlation between them (*r*_*S*_ (9) = 0.39, *p* = 0.236 for RJA, and *r*_*S*_ (9) = 0.30, *p* = 0.377 for RNA). On the other hand, there was a strong statistically significant correlation between the two robot-assessed features (*r*_*S*_ (9) = 0.63*, *p* = 0.037) and between the two human-assessed features (*r*_*S*_ (9) = 0.66*, *p* = 0.026).

These results show that children’s response to the human-administered ADOS tasks do not directly correlate to their response to a similar interaction with a robot, but that the cross-task response relationship is maintained. Moreover, the children overall needed significantly higher action levels with the robot than when interacting with a human (*M* = 2.55 versus *M* = 1.45 for RJA, and *M* = 2.09 versus *M* = 1.64 for RNA). In particular, on the one hand, the human-assessed values didn’t exceed a value of 2, which is most likely indirectly due to our selection criteria favoring enough attention span to follow a story (a criteria which seems to correlate with our feature values since both are related to attention). In contrast, on the other hand, the robot-assessed RJA values spanned all 4 possible values, which means that children where less sensitive to the robot’s stimuli in comparison with the human’s.

In addition to RJA and RNA, an autism expert also coded the response to the four imitation prompts performed by the robot throughout the story. For each prompt, she coded the response into three ordinal categories (satisfactory 1), below average 2), and poor 3)). We then aggregated these responses into a single feature for each child according to Eq. 1. A Spearman correlation test showed a strong and statistically significant correlation between the response to the robot’s imitation prompts and the robot-assessed RJA (*r*_*S*_ (8) = 0.73*, *p* = 0.016), as well as the robot-assessed RNA (*r*_*S*_ (8) = 0.66*, *p* = 0.037).

In the ADOS, there is no feature specifically dedicated to response to imitation prompts (although there is a task that revolves around functional and symbolic imitation). Therefore, before the session the examiner who performed the reassessment of RJA and RNA also assessed imitation ability. She simply performed one of the prompts the robot would perform (namely, the “Running” prompt) and asked the child to imitate her. She then coded the response in the same three categories as above. The response to robot-prompted imitation was not found to be statistically significant correlated with the response to human-prompted imitation (*r*_*S*_ (8) = 0.40, *p* = 0.254). Similarly to the results with RJA and RNA, children had statistically significantly poorer response on imitation with the robot than with the human, as shown by a Wilcoxon signed rank test (*Z* < 0.001*, *p* = 0.015). Interestingly, the response to human-prompted imitation correlated with both the robot-assessed RJA (*r*_*S*_ (8) = 0.73*, *p* = 0.016) and with the robot-assessed RNA (*r*_*S*_ (8) = 0.66*, *p* = 0.037).

### 6.3 Comparison of Modes

In analyzing the occurrence of successes across the different modes, we used four different metrics:

• *Within-4 success rate*—i.e., the percentage of task instances in which a success occurred within the exhaustion of a full action sequence (i.e., at most 4 trials).
• *Within-2 success rate*—i.e., the percentage of instances in which a success occurred within at most two trials of an action sequence.
• *Average number of trials*—i.e., the average number of actions the robot had to execute during a task instance.
• *Average successful action level*—i.e., the average level of all actions that caused a success.

To illustrate the computation of these metrics using the example of Figure 3 for a single interaction, the Therapy mode shows successes after trials 2, 1, none, and 3, for the first, second, third, and fourth instances of JATT in that mode respectively. As a result, the within-4 success rate is 3/4 = 75%, the within-2 success rate is 2/4 = 50%, the average number of trials is (2 + 1+5 + 3)/4 = 2.75 (no success after 4 trials is treated as a 5), and the average successful level is (3 + 3+4)/3 = 3.33 (no success after 4 trials ignored).

The results for these four metrics in our study across all participants are reported in Table 2. Our comparative analysis of the different modes showed that our sample was not large enough to achieve statistical significance on most of the pairwise comparisons of modes for the different metrics, as evaluated by both a repeated measures and a mixed-effects model (*p* > 0.05). Therefore, we advise the reader to take the following analysis merely as suggestive comparative results to guide further investigation.

**TABLE 2:** Comparison of success occurrences in the three modes.

Metric	Assess (*n* = 44)		Therapy (*n* = 40)		Explore (*n* = 40)	
	JATT/NAME	Total	JATT/NAME	Total	JATT/NAME	Total
Within-4 success (%)	97.5/100	98.8	100/72.5	86.3	100/87.5	93.4
Within-2 success (%)	70.0/77.5	73.4	80.0/62.5	71.3	97.5/65.0	81.3
Average #(trials)	2.40/2.00	2.20	1.55/2.45	2.00	1.25/1.98	1.62
Average successful level	2.43/1.95	2.19	2.58/2.18	2.38	2.70/2.64	2.67

Overall, the Assess mode required the lowest action level on average to achieve a success, but at the cost of the highest average number of trials. It also had the highest within-4 success rate, which can be explained by the fact that it was ensuring that the children were exposed to a maximum number of different actions. The Therapy mode needed lower action levels to achieve a success on average as compared to the Explore mode, but higher than the Assess mode. However it needed less trials than the Assess mode, and more than the Explore mode. On the other hand, it had the lowest success rate on both metrics, except for task JATT where it outperformed mode Assess. The Explore mode had the highest within-2 success rate and the lowest average number of trials, but at the cost of needing the highest action level on average to achieve a success.

Since cross-task comparisons in the Assess mode were previously included, we focus our cross-task analysis here on modes Therapy and Explore. In both these modes, the JATT task showed a success rate above 80% across the first two metrics. The NAME task, however, showed significantly lower success rate, according to a two-proportion Z test, both within four trials (*Z* = 8.94**, *p* < 0.01), as well as within two trials (*Z* = 7.69**, *p* < 0.01). Similarly, a Wilcoxon signed rank test showed that the median average number of trials per participant was significantly lower for the JATT task as compared to the NAME task for both Therapy mode (*Z* = 36.00*, *p* = 0.011) and Explore mode (*Z* = 28.00*, *p* = 0.018). This same test showed no statistically significant results when considering the average successful level per participant (*Z* = 7.00, *p* = 0.236 for mode Therapy and *Z* = 24.00, *p* = 0.857 for mode Explore).

Finally, it is important to stress that a comparison of the different modes in a single session does not provide any information about the long-term benefits of these modes. This comparison is merely informative of how sequencing affects the children’s response along the static metrics we selected. The session was too short to expect any practice effects, and we did not find any evidence of such effects in our data. We reported all three modes in the table, although it is to be noted that a methodologically sound comparison can only be made between modes Therapy and Explore, since several scenario-related factors differ in the Assess mode.

## 7 Discussion

The results of the study bring insight into the structure of children profiles, the effect of action sequencing on children’s responses, and differences between tasks. We discuss each of these in turn, and end this section with additional thoughts.

### 7.1 Children Profiles

Our comparison of profiles in the interaction with the robot versus with a human suggests that the information encoded in the human-administered ADOS cannot be used directly to inform an interaction with a robot. In addition to the lack of evidence for a correlation between the two, the children overall needed higher action levels with the robot than when interacting with a human. This result is in accordance with existing literature on socially assistive robotics (Anzalone et al., 2014; Warren et al., 2015).

This result can be explained by the lower degree of expressivity and naturalness of the robot as compared to a human, or by the lack of familiarity of the children with the robot’s behavior. In particular when it comes to gaze, literature on general human response to robot gaze has also shown reduced reflexive gaze as compared to response to human gaze, which may have been a contributing factor in our JATT task (Admoni et al., 2011). It is also worth mentioning that the robot performed each action only once, while in the context of the ADOS actions are repeated several times to ensure a lack of response at a given level before moving to the next. In our scenario, we eliminated repetitions because we expected a very high number of trials to be harmful for engagement. However, we collected several measurements to reduce the effect of the incurred noise. These observations highlight the importance of having the robot perform its own assessment to be able to model the children’s responses to its own actions accurately, and ensure the validity of personalized robot intervention such as in the Therapy mode.

On the other hand, our data showed significant correlations among robot-assessed features, including response to imitation prompts. These results may have implications on the development of more efficient methods for co-estimating those correlated variables, or for predicting cross-task performance from measurements on a single task.

### 7.2 Effect of Sequencing

In this exploratory study, we analyzed the effect of sequencing on child response through controlling the robot mode. Based on our analysis, we observed that each of the modes comes with advantages and disadvantages.

First, the Assess mode favors using as low action levels as possible to cause a success. Based on our results, it seems to be well suited, beyond assessment, for cases where therapeutic goals need to be met with no concern for minimizing the number of trials. This applies when engagement and interaction flow are not priorities, for example in scenarios in which the tasks are repeated only a small number of times, or are sparsely distributed in time.

On the other extreme, the Explore mode seems to be a suitable mode if the only goal is to achieve early successes and to keep the child as engaged as possible. Its surprisingly relatively high success rate, especially as compared to the Therapy mode, may be due to the high level of variability in action levels, which may cause children to respond more frequently. This effect could be explained by the existing literature on how statistically “surprising” events lead to higher attention responses (Itti and Baldi 2006; Klein et al., 2019). Despite its higher success rate, the Explore mode does not align with therapeutic principles of grading and just-right challenge characterized by consistency, scaffolding and gradual change in actions (Hersch et al., 2005; Schaaf and Roley 2006). As a result, it would not be suitable to be used for therapeutic purposes whose aim is to promote a positive change in response over time. Between these two extremes, the Therapy mode aims to balance causing successes at low action levels and preferring a smaller number of trials in a personalized way, according to assessed children profiles.

In short-term studies the novelty factor of the robot may have a strong effect on child response and may not reflect actual characteristics of the disorder, because as has been demonstrated in this work, the response to the robot greatly varied, regardless of ASD severity. In long-term studies, the novelty effect may disappear. In contrast, the engagement of the child may also decrease, so long-term studies should be looked at with care.

Linking back to our initial motivation on the need for personalized robot behavior, our study suggests that an “optimal” personalized action sequence (in terms of balancing different interaction goals) could take the Therapy mode as a baseline and iterate on it using additional data to create more powerful algorithms for automatic action sequence generation. On the other hand, our results also highlight that different action sequences should be selected based on context, possibly in response to indicators such as affect, engagement, and progress during therapy. The results presented in this paper provides future research in this space with some decision-making guidelines based on the aim and scope of personalization in therapeutic interventions.

### 7.3 Cross-Task Differences

There are a few possible explanations as to why we observed cross-task differences across modes that were not consistent with the human-assessed children profiles. These include the objective difficulty of the prompts, the nature of the scenario, the relative interest of the children in the cartoons versus the robot, and the relative cartoon novelty as compared to the consistency of the robot’s appearance. Identifying the exact causes or combinations of causes would need additional research.

Since the tasks we considered are quite generic and can be easily adapted to a range of different scenarios with different targets, we expect good generalizability of our general findings across similar scenarios. For example, any target object can be equipped with controllable lights and sound, to play the role of video and sound from our scenario, and we expect similar response patterns to hold across classes of similar tasks.

### 7.4 Robotic Platform

The use of a NAO humanoid robot provided both advantages and disadvantages for the research goals of this work. On the positive side, the embodiment and size of the robot make it generally attractive to children with ASD, as demonstrated by several studies (Tapus et al., 2012; Anzalone et al., 2014; Greczek et al., 2014; Yussof et al., 2015; Esteban et al., 2017) and confirmed in ours. Moreover, its humanoid appearance and control of individual joints allowed for flexible gesturing options, while using speech and lights as additional expressive modalities. The multi-modal aspect of the communication was especially useful to allow the progressive integration of social cues throughout the action scales, and contributed to keeping the children engaged. On the negative side, the robot lacked actuated eyes, which limited the expressivity of the robot’s gaze behavior. Furthermore, its speech was often monotonous or unclear despite our best efforts to make it engaging and articulate.

While we expect our general results to hold across different kinds of humanoid platforms, we hypothesize that the degree of anthropomorphism of the robot will have an effect on both the success rate of individual actions, and the discrepancy between the children’s response to the human versus the robot. For example, we expect more responsiveness and less discrepancy between human-assessed and robot-assessed features for more anthropomorphic robots (e.g., robots with eyes, hair, or artificial skin). Additional research is needed to verify this hypothesis.

### 7.5 Other Observations and Lessons Learnt

We observed that the first contact with the robot was crucial in determining if the child will accept or refuse to interact with the robot. Several of our pilot attempts failed because of lack of care during this critical phase, which we corrected for the actual study. For future research, we highlight the need to very progressively integrate communication modalities to avoid negative reactions of the child in first-time encounters with a robot.

Children with ASD are a particularly challenging population to work with, as the slightest change in stimulus can cause a large difference in outcome. For example, the way the screens were flashing had a big influence on whether the children responded or not, so a lot of care had to be put in fine-tuning the scenario parameters during our pilots. These parameters included the screen behavior, the volume of the robot, the placement of the screens, the story length and content, the interval between consecutive task instances, the positioning of the imitation prompts, among other considerations that we iteratively refined.

Also, the variability across children also affected other aspects beyond the performance on the tasks. For example, while some children paid full attention to the story and were fascinated by the robot’s behavior, others had moments of complete distraction. Such distraction moments included being fascinated by aspects of the interaction irrelevant to the story, like for instance fixating or touching a body part of the robot.

A final observation concerns a hypothesized relationship between engagement and practice effects. While there is no quantitative evidence of a consistent change in behavior throughout the session, we noticed that two effects seemed to balance each other differently across children. On the one hand, we noticed that engagement tends to plateau after a few minutes of interaction and then starts decreasing towards the last third of the session, which may worsen the performance of children on the tasks in that period. On the other hand, practice effects have the opposite effect level on task performance in that they increase it. Therefore, it is difficult to dissociate these two effects in our data, and future research could look at examining them separately to further inform robot adaptation within the session.

## 8 Conclusion and Future Work

The goal of this paper was to study how different ways of sequencing a socially assistive robot’s actions can affect the behavioral responses of a child with ASD. We studied this problem in the context of two robot-assisted attention-related therapy tasks inspired by the ADOS diagnostic tool. In a first step, we leveraged the structure of the ADOS tasks to build robotic actions on the NAO robot. We then integrated those actions into a control architecture that allows the robot to operate in three modes: Assess, Therapy, and Explore. These modes generate different sequences of the same robot actions, with different properties. To evaluate the effect of the different modes, we developed a semi-autonomous robotic scenario based on interactive storytelling, which integrates the tasks.

Our data collected with 11 children with different ASD severities highlight the advantages and disadvantages of each mode depending on the interaction goals. The Assess mode favored a consistent and progressive evolution of action levels and had the highest therapeutic value, at the cost of a relatively high number of trials. The Explore mode had the lowest number of trials but the least therapeutic value. The Therapy mode found a tradeoff between meeting the conflicting goals of maximizing therapeutic value while minimizing number of trials. The presented differences are however not statistically significant, but based on the descriptive values on our selected metrics. To confirm this interpretation, replication studies with higher sample sizes are needed.

Based on these preliminary results, we propose the following guidelines. The choice of robot action sequences in socially assistive tasks should be made (whether autonomously or by design) in accordance to the goals of the interaction. If the interaction purely aims at challenging the child to learn, then a consistent and progressive action sequence (such as mode Assess) should be preferred. On the other end of the spectrum, if the interaction aims at maintaining engagement and fluency, then a high-variability action sequence (such as mode Explore) should be preferred. Finally, if the interaction aims to find a tradeoff between those conflicting goals for an optimal learning experience, then a personalized and progressive action sequence (such as Therapy) should be preferred, although it is unclear based on this study what such an optimal action sequence would be.

To follow up on this question, the data collected in this work has been used to automatically generate action sequences following a decision-theoretic formalism (Baraka et al., 2020). These action sequences are aimed at providing a “just-right challenge” for an optimal learning experience. Our model assumes probabilities of success for each action (based on the interaction data collected in this study) and costs for each action (based on a survey with experts). Based on this model, our algorithm computes sequences that optimize for an objective function that includes these two components.

A potential further extension of the personalization approach described in this manuscript would be to introduce mechanisms to re-evaluate or adjust the robot’s behavior in real-time according to the child’s response, to account for variations in engagement and abilities over time. In addition, further personalization of individual actions would be possible based on individual preferences or needs (e.g., adjusting light colors or motion speed, avoiding behaviors that are scary or triggering for a particular child, etc.).

While the study presented in this article focused on ASD children and behavior-centric interventions, we expect our high-level results to hold in other similar contexts aimed to induce a change in the human over time, such as educational contexts and other types of tasks (e.g., cognitive) whose difficulty level can easily be controlled. Future empirical work could include studying the long-term effects of different action sequences to inform the development of long-term optimal robot control policies that balance engagement and learning goals, bringing us a step closer towards flexible and personalized intelligent robotic tools for richer and more effective behavioral interventions.

## Data Availability

The datasets presented in this article are not readily available because only aggregate data can be made available publicly, according to ethics committee's approval. Requests to access the datasets should be directed to kbaraka@alumni.cmu.edu.
